# Hemodynamic Improvement With the Double-Tap Technique During Transcatheter Aortic Valve Replacement Using a Pressure-Sensing Guidewire

**DOI:** 10.1016/j.jaccas.2026.107860

**Published:** 2026-04-12

**Authors:** Yohei Nishimura, Kengo Tanabe, Akihiro Higashino, Motoi Yokozuka, Yu Horiuchi, Masanori Taniwaki, Hitomi Yuzawa, Kota Komiyama, Jun Tanaka, Masahiko Asami

**Affiliations:** a Division of Cardiology, Mitsui Memorial Hospital, Tokyo, Japan; b Department of Cardiovascular Surgery, Mitsui Memorial Hospital, Tokyo, Japan; c Division of Anesthesia, Mitsui Memorial Hospital, Tokyo, Japan

**Keywords:** balloon-expandable valve, postdilatation, SAPIEN 3, SavvyWire, transcatheter aortic valve implantation (TAVI)

## Abstract

Postdilatation using the original delivery system balloon at the same filling volume (the double-tap technique) has been proposed to improve balloon-expandable transcatheter heart valve (THV) expansion during transcatheter aortic valve replacement (TAVR); however, its immediate hemodynamic impact remains unclear. Here, we report a case series of 6 consecutive patients who underwent TAVR with balloon-expandable valves in whom the double-tap technique was performed with hemodynamic assessment using a pressure-sensing guidewire. The mean transvalvular pressure gradients and midportion THV diameters were evaluated before and after the double-tap technique. This technique was significantly associated with reduced mean transvalvular pressure gradients (median difference: −6.5 mm Hg; *P* = 0.036) and increased midportion THV diameter (median difference: 0.8 mm; *P* = 0.036), whereas paravalvular leak was reduced to trivial or none in all patients, and no cardiovascular death, stroke, or permanent pacemaker implantation occurred at 30 days. The double-tap technique under pressure-sensing guidewire guidance may facilitate safer valve optimization during TAVR.

## Visual Summary

**Figure:**
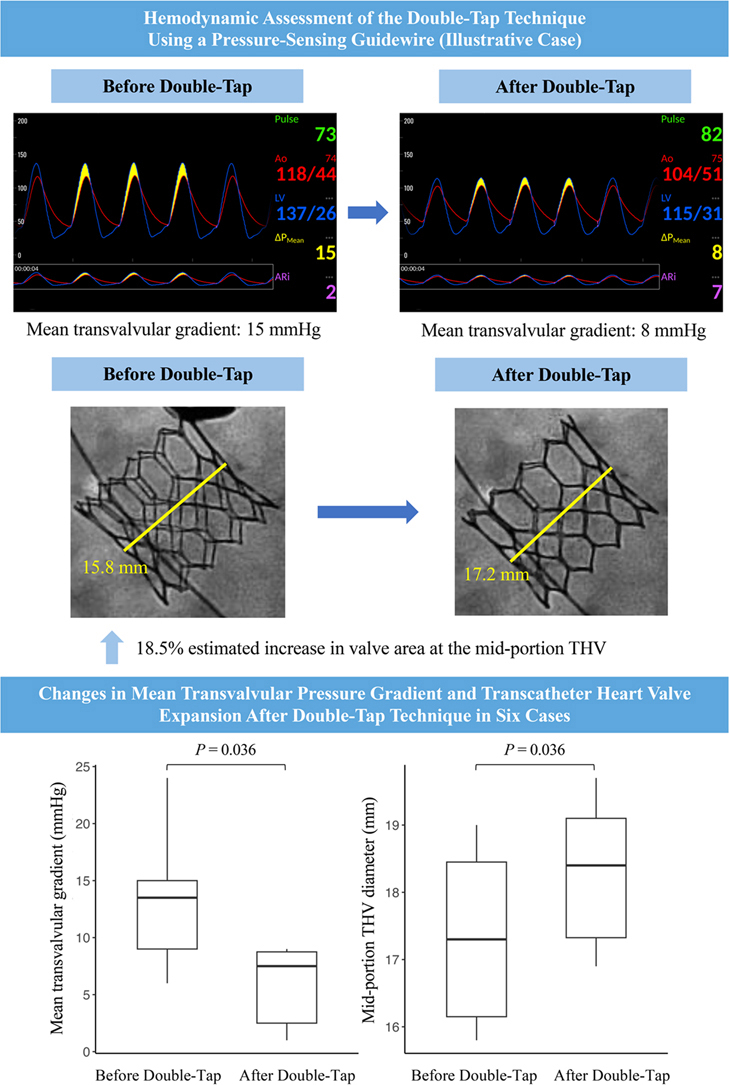

Postdilatation using the original delivery system balloon at the same filling volume (the double-tap technique) in patients undergoing transcatheter aortic valve replacement (TAVR) with balloon-expandable valves (BEVs) has recently emerged as a simple strategy to improve transcatheter heart valve (THV) expansion without the need for balloon overfilling or additional valvuloplasty balloons.^1^^,^^2^ Previous studies have evaluated the double-tap technique using fluoroscopic morphology; however, its hemodynamic impact remains unknown.

SavvyWire (OpSens Medical), a pressure-sensing guidewire capable of continuous beat-to-beat hemodynamic assessment during valve deployment,^3^ provides an opportunity to directly quantify the physiological effect of postdilatation. Here, we report a consecutive case series in which SavvyWire was used to assess the real-time hemodynamic response to the double-tap technique after TAVR with BEVs.

## Case Presentation

### Patient 1

An 85-year-old woman with a history of essential hypertension and newly diagnosed severe aortic stenosis (AS) was admitted for acute decompensated heart failure. After symptom improvement with intravenous diuretic agents, she underwent transfemoral TAVR with a 23-mm SAPIEN 3 Ultra RESILIA (S3UR) valve (Edwards Lifesciences). Preprocedural computed tomography (CT) demonstrated an aortic valve calcium score of 2,557 Agatston units (AU). After the initial deployment, SavvyWire demonstrated a persistent mean transvalvular pressure gradient (MPG) of 15 mm Hg. The double-tap technique was performed using the original delivery system balloon with the same filling volume, resulting in an immediate reduction in the MPG to 9 mm Hg and an increase in the midportion THV diameter from 16.3 to 17.7 mm, corresponding to a 17.9% increase in valve area at the midportion of the THV (Supplemental Figure 1). After the initial valve deployment, the paravalvular leak (PVL) was trivial and resolved to none.

### Patient 2

An 81-year-old woman with a history of essential hypertension and dyslipidemia presented with exertional dyspnea. Echocardiography confirmed symptomatic severe AS. She underwent transfemoral TAVR with a 23-mm S3UR valve. Preprocedural CT revealed an aortic valve calcium score of 1,629 AUs. After the initial valve deployment, the MPG remained elevated (15 mm Hg) on SavvyWire assessment. No PVL was observed after the initial valve deployment. The double-tap technique was performed, resulting in an immediate reduction in the MPG to 8 mm Hg and an 18.5% increase in the valve area at the midportion of the THV (Supplemental Figure 2).

### Patient 3

An 88-year-old woman with a history of percutaneous coronary intervention was followed for moderate AS. She developed exertional dyspnea, and repeat coronary angiography demonstrated no in-stent restenosis. However, AS progressed in severity, prompting transfemoral TAVR with a 20-mm S3UR valve. Preprocedural CT demonstrated an aortic valve calcium score of 1,194 AUs. After the initial valve deployment, the MPG remained elevated (24 mm Hg) on SavvyWire assessment. The double-tap technique was performed, resulting in an immediate reduction in the MPG to 7 mm Hg and a 10.2% increase in the valve area at the midportion of the THV (Supplemental Figure 3). After the initial valve deployment, the PVL was mild and resolved to none.

### Patient 4

An 82-year-old man with a history of atrial fibrillation and ischemic stroke was admitted to another hospital for acute decompensated heart failure. After decongestion, the patient was transferred to our institution for definitive management of severe AS and underwent transfemoral TAVR with a 23-mm S3UR valve. Preprocedural CT revealed an aortic valve calcium score of 2,959 AUs. After the initial valve deployment, the MPG remained elevated (12 mm Hg) on SavvyWire assessment. The double-tap technique was performed, resulting in an immediate reduction in the MPG to 9 mm Hg and a 6.6% increase in the valve area at the midportion of the THV (Supplemental Figure 4). After the initial valve deployment, the PVL was mild and resolved to none.

### Patient 5

A 90-year-old woman with a history of moderate pulmonary artery enlargement presented with exertional dyspnea. Transthoracic echocardiography revealed severe AS, and the patient was referred to our hospital. On the basis of clinical symptoms and echocardiographic findings, transfemoral TAVR with a 23-mm S3UR valve was performed. Preprocedural CT demonstrated an aortic valve calcium score of 2,108 AUs. After the initial valve deployment, the MPG remained elevated (8 mm Hg) on SavvyWire assessment. The double-tap technique was performed, resulting in an immediate reduction in the MPG to <1 mm Hg and a 7.5% increase in the valve area at the midportion of the THV (Supplemental Figure 5). After the initial valve deployment, the PVL was trivial and remained trivial after this technique.

### Patient 6

A 78-year-old woman was referred after the detection of a cardiac murmur during a routine health checkup. Transthoracic echocardiography demonstrated severe AS, and the patient reported exertional dyspnea. Considering the symptomatic status and severity of valve disease, transfemoral TAVR with a 23-mm S3UR valve was selected. Preprocedural CT revealed an aortic valve calcium score of 1,088 AUs. After the initial valve deployment, the MPG remained elevated (6 mm Hg) on SavvyWire assessment. The double-tap technique was performed, resulting in an immediate reduction in the MPG to <1 mm Hg and an 8.9% increase in the valve area at the midportion of the THV (Supplemental Figure 6). After the initial valve deployment, the PVL was trivial and resolved to none.

### Summary of the case series

This case series included 6 consecutive patients with severe AS who underwent TAVR with BEVs in whom a routine double-tap technique and hemodynamic assessment using a pressure-sensing guidewire were performed in all cases. At our institution, the double-tap technique was routinely performed in all balloon-expandable TAVR procedures without predefined hemodynamic or fluoroscopic criteria. After standard aortic valve crossing, a pigtail catheter was positioned in the left ventricular (LV) apex, and the pressure-sensing guidewire was zeroed and advanced through it to enable continuous invasive hemodynamic monitoring. Simultaneous LV-aortic pressures were recorded throughout the procedure. During valve deployment and the double-tap technique, the guidewire was maintained at the LV apex, with fluoroscopic confirmation of the tip position before each inflation. The guidewire position was also intermittently confirmed using intraoperative transesophageal echocardiography, and correct placement at the LV apex was verified in all cases before pressure measurements. No repositioning was required in any case. Baseline characteristics and procedural details are summarized in Table 1. SavvyWire-measured MPG, midportion THV diameter, aortic regurgitation (AR) index, and transesophageal echocardiographic–assessed AR severity were assessed before and after the double-tap technique. The estimated percentage of increase in the midportion THV area was calculated for each patient. THV frame expansion was evaluated fluoroscopically using the commissural post height method,^4^ and the midportion THV diameter was used as a practical surrogate for valve expansion.^1^^,^^5^ Quantitative assessment of THV frame expansion was performed retrospectively using imaging analysis for exploratory purposes. No patients experienced cardiovascular death, stroke, or permanent pacemaker implantation at 30 days.

**Table 1:** Summarization of Patients Undergoing TAVR Using the Double-Tap Technique

Patient No.	Age (y)	Sex	Aortic Valve Calcium Score (AU)	Valve Type		SavvyWire MPG (mm Hg)	Midportion THV Diameter (mm)	Estimated Increase of Midportion THV Area (%)	AR Index	Transesophageal AR Severity	30-day Outcomes
1	85	Female	2,557	S3UR 23 mm	Before double tap^a^	15	16.3	–	37	Trivial	No complications^b^
					After double tap	9	17.7	17.9	37	None	
2	81	Female	1,629	S3UR 23 mm	Before double tap^a^	15	15.8	–	2	None	No complications^b^
					After double tap	8	17.2	18.5	7	None	
3	88	Female	1,194	S3UR 20 mm	Before double tap^a^	24	16.1	–	20	Mild	No complications^b^
					After double tap	7	16.9	10.2	6	None	
4	82	Male	2,959	S3UR 23 mm	Before double tap^a^	12	18.5	–	27	Mild	No complications^b^
					After double tap	9	19.1	6.6	17	None	
5	90	Female	2,108	S3UR 23 mm	Before double tap^a^	8	19.0	–	16	Trivial	No complications^b^
					After double tap	<1	19.7	7.5	16	Trivial	
6	78	Female	1,088	S3UR 23 mm	Before double tap^a^	6	18.3	–	25	Trivial	No complications^b^
					After double tap	<1	19.1	8.9	17	None	

AR = aortic regurgitation; AU = Agatston unit; MPG = mean pressure gradient; TAVR = transcatheter aortic valve replacement; THV = transcatheter heart valve; S3UR = SAPIEN 3 Ultra RESILIA.

a “Before double tap” refers to measurements obtained after initial valve deployment and before the double-tap technique.

b Complications include cardiovascular death, stroke, or permanent pacemaker implantation.

**Figure 1:**
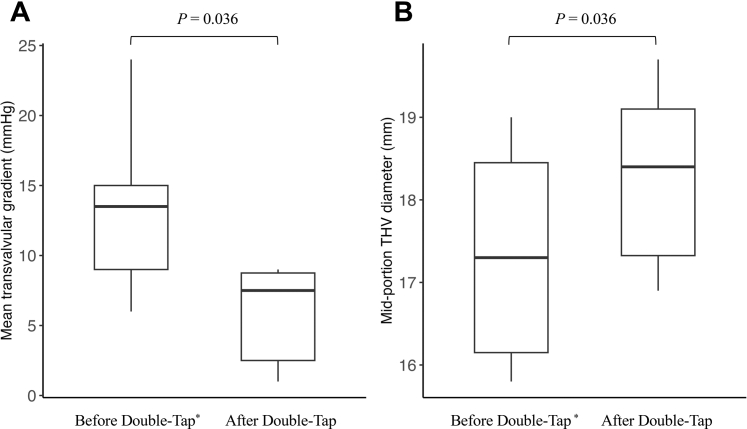
Hemodynamic and Geometric Changes After the Double-Tap Technique Box-and-whisker plots of the (A) mean pressure gradient and (B) midportion transcatheter heart valve (THV) diameter before and after the double-tap technique. A paired Wilcoxon signed rank test is performed. ∗“Before double-tap” refers to measurements obtained after initial valve deployment and before the double-tap technique.

In all cases, morphological improvement of the valve frame and functional improvement in hemodynamics were observed after the double-tap technique (Table 2, Figures 1A and 1B). The MPG were significantly reduced (median difference: −6.5 mm Hg; Wilcoxon signed rank test: *P* = 0.036), and the midportion THV diameter significantly increased (median difference: 0.8 mm; *P* = 0.036). The estimated midportion THV area increased in all patients, with a median increase of 9.6% (*P* = 0.036). No significant change in the AR index was observed (median difference: −4.0 mm Hg; *P* = 0.201). After valve deployment, AR severity ranged from mild to none; after the double-tap technique, AR resolved completely in 4 patients, with trivial residual regurgitation in 1 patient. One patient had no AR both before and after this technique.

**Table 2:** Hemodynamic and Geometric Changes After the Double-Tap Technique

	Before Double Tap^a^	After Double Tap	Median Difference (Post − Pre)	*P* Value
SavvyWire MPG, mm Hg	13.5 (9.0-15.0)	7.5 (2.5-8.8)	−6.5	0.036
Midportion THV diameter, mm	17.3 (16.2-18.5)	18.4 (17.3-19.1)	0.8	0.036
Estimated midportion THV area, mm^2^	235.9 (203.6-268.8)	266.3 (224.3-286.5)	22.4	0.036
AR index	22.5 (17.0-26.5)	16.5 (9.3-17.0)	−4.0	0.201

Values are median (IQR).

Abbreviations as in Table 1.

a “Before double tap” refers to measurements obtained after initial valve deployment and before the double-tap technique.

## Discussion

In this consecutive clinical case series of 6 patients undergoing TAVR with BEVs, the principal findings of this case series were as follows: 1) postdilatation using the original delivery system balloon was associated with an immediate reduction in the MPG, as assessed by real-time intraprocedural pressure monitoring; 2) hemodynamic improvement was observed alongside the expansion of the midportion of the THV frame; and 3) physiological and morphological improvements occurred without adverse early safety outcomes.

Underexpansion of the THV, as assessed by the midportion THV diameter, has been associated with increased risk of hemodynamic valve deterioration.^5^ Additionally, underexpansion and deformation of THV frames have been implicated in adverse leaflet biomechanics, including increased leaflet stress, hypoattenuating leaflet thickening, and potentially reduced valve durability.^6^^,^^7^ Analogous observations in percutaneous coronary intervention suggest that multiple, short postdilatations can achieve superior expansion compared with a single prolonged inflation,^8^ supporting the rationale for repeated balloon inflations. Consistent with this concept, routine postdilatation during TAVR, compared with local standard practice, has been reported to improve the MPG and reduce prosthesis-patient mismatch at 30 days without compromising procedural success,^9^ supporting its potential clinical use. However, concerns remain regarding the need for balloon overfilling or the use of additional valvuloplasty balloons. A previous study reported that the routine double-tap technique improves frame expansion based on fluoroscopic assessment, with no stroke or cardiovascular mortality reported at 30 days.^2^ Recent data evaluating routine double-tap technique with the latest-generation BEVs (S3UR) similarly demonstrated significant improvements in frame expansion and foreshortening without early safety concerns.^10^ However, these analyses were primarily based on fluoroscopic measurements, and the direct physiological impact of postdilatation has remained less well characterized. In this context, the present study extends these findings by demonstrating immediate invasive hemodynamic improvement using real-time pressure monitoring. To the best of our knowledge, this is the first case series to document its immediate physiological effects using real-time pressure monitoring with SavvyWire.

The double-tap technique was associated with improved midframe expansion and reduced MPG, in line with previous studies suggesting that this technique improves THV underexpansion without any adverse early clinical outcomes. Notably, in the present case series, the estimated increase in the midportion THV area was <10% in several patients and did not appear to be remarkable from a purely morphologic standpoint; however, measurable hemodynamic improvement was consistently observed. The immediate and consistent hemodynamic response indicates that minor geometric optimization, which is often considered clinically trivial based on fluoroscopic appearance alone, may influence transvalvular hemodynamics.

From a clinical perspective, the double-tap technique may offer a safe and pragmatic means of improving transvalvular hemodynamics without balloon overfilling, additional catheter exchange, or additional valvuloplasty balloons. Most strokes associated with TAVR occur in the periprocedural period, particularly within the first 48 hours, and are believed to be predominantly embolic in nature, related to catheter and device manipulation in the setting of atherosclerotic and calcified aortic anatomy.^11^^,^^12^ Simplification of postdilatation with the double-tap technique may be conceptually associated with less catheter and balloon manipulation, potentially reducing aortic plaque disturbance during balloon delivery. Importantly, immediate hemodynamic assessment using a pressure-sensing guidewire provides objective feedback that complements fluoroscopic assessment with additive functional information. Such integration warrants further investigation and may help identify patients who will derive meaningful hemodynamic benefits while avoiding unnecessary additional interventions.

In this case series, the double-tap technique was applied routinely during the study period. However, the present analysis was not designed to determine whether routine application offers advantages over a more selective approach. Future studies are required to clarify whether the double-tap technique should be systematically performed or selectively used in cases with residual gradients or suspected valve underexpansion. None of the 6 patients experienced severe adverse events, suggesting that the routine double-tap technique during TAVR may be a reasonable option for optimizing valve performance. Nonetheless, this single-center case series should be considered hypothesis generating. Transesophageal echocardiography–derived Doppler gradients were not systematically obtained in this case series because Doppler-based gradient assessment after balloon-expandable TAVR is not routinely performed at our institution because of potential overestimation of the true transvalvular gradient due to pressure recovery.^13^ Although prior studies have demonstrated good agreement between pressure wire and catheter-based measurements in the TAVR setting,^14^ the absence of multimodality validation represents a limitation of the present study. The long-term clinical significance of the observed hemodynamic and geometric improvements has not been established because the lack of a control group and absence of long-term follow-up limit the interpretability of these findings. Further studies are warranted to address these limitations.

## Conclusions

Based on these 6 cases, the double-tap technique during TAVR with BEVs appears to be a feasible and safe option for optimizing valve performance, with improved THV expansion and an immediate reduction in the MPG. The integration of a pressure-sensing guidewire may enable safer and more efficient optimization of valve performance during TAVR. However, given the small sample size and absence of a comparator group, these findings should be considered hypothesis generating and require validation in larger prospective studies.

**Visual Summary:**
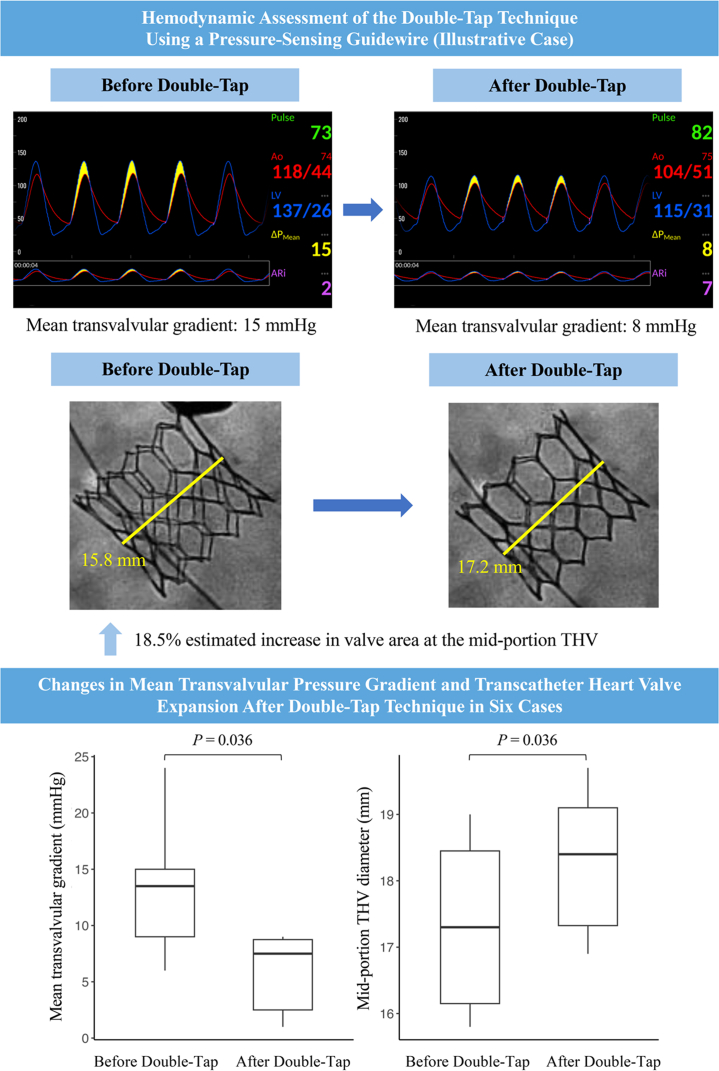
Hemodynamic and Geometric Effects of the Double-Tap Technique Assessed by a Pressure-Sensing Guidewire Representative pressure waveforms obtained with a pressure-sensing guidewire (SavvyWire) and corresponding fluoroscopic images from patient 2 demonstrate reduced mean pressure gradient and improved midportion transcatheter heart valve (THV) expansion before and after the double-tap technique (top and middle). In this representative case, the mean pressure gradient decreased from 15 to 8 mm Hg, with an 18.5% increase in the midportion THV area. Box-and-whisker plots summarize the changes in mean pressure gradient and midportion THV diameters across all 6 cases (bottom). Paired comparisons are performed using the Wilcoxon signed rank tests.

## Funding Support and Author Disclosures

Dr Tanabe has received honoraria from Abbott Medical, Boston Scientific, Edwards Lifesciences, and Medtronic, outside of the submitted work. Dr Higashino has received honoraria from Abbott Medical, outside of the submitted work. Dr Taniwaki has received honoraria from Abbott Medical and Boston Scientific, outside of the submitted work. Dr Yuzawa has received honoraria from Boston Scientific and Medtronic, outside of the submitted work. Dr Komiyama has received honoraria from Abbott Medical, outside of the submitted work. Dr Tanaka has received honoraria from Abbott Medical, Boston Scientific, and Edwards Lifesciences, outside of the submitted work. Dr Asami serves as a clinical proctor for Abbott Medical, Edwards Lifesciences, and Medtronic; and has received honoraria from Abbott Medical, Boston Scientific, Edwards Lifesciences, and Medtronic, outside of the submitted work. All other authors have reported that they have no relationships relevant to the contents of this paper to disclose.

**Take-Home Messages**

• The double-tap technique during TAVR using BEVs was associated with THV expansion and an immediate reduction in mean transvalvular pressure gradient.
• The integration of a pressure-sensing guidewire with the double-tap technique may enable safer and more efficient optimization of valve performance during TAVR.
