# Supplementary material for: Hemodynamic Improvement With the Double-Tap Technique During Transcatheter Aortic Valve Replacement Using a Pressure-Sensing Guidewire
Source: JACC Case Rep. 2026 Apr 12;31(21):107860. doi: 10.1016/j.jaccas.2026.107860 (PMC13221822; doi:10.1016/j.jaccas.2026.107860)

**Supplementary Figures: Hemodynamic Assessment of the Double-tap Technique During Transcatheter Aortic Valve Replacement Using a Pressure-sensing Guidewire**

Supplementary Figure 1


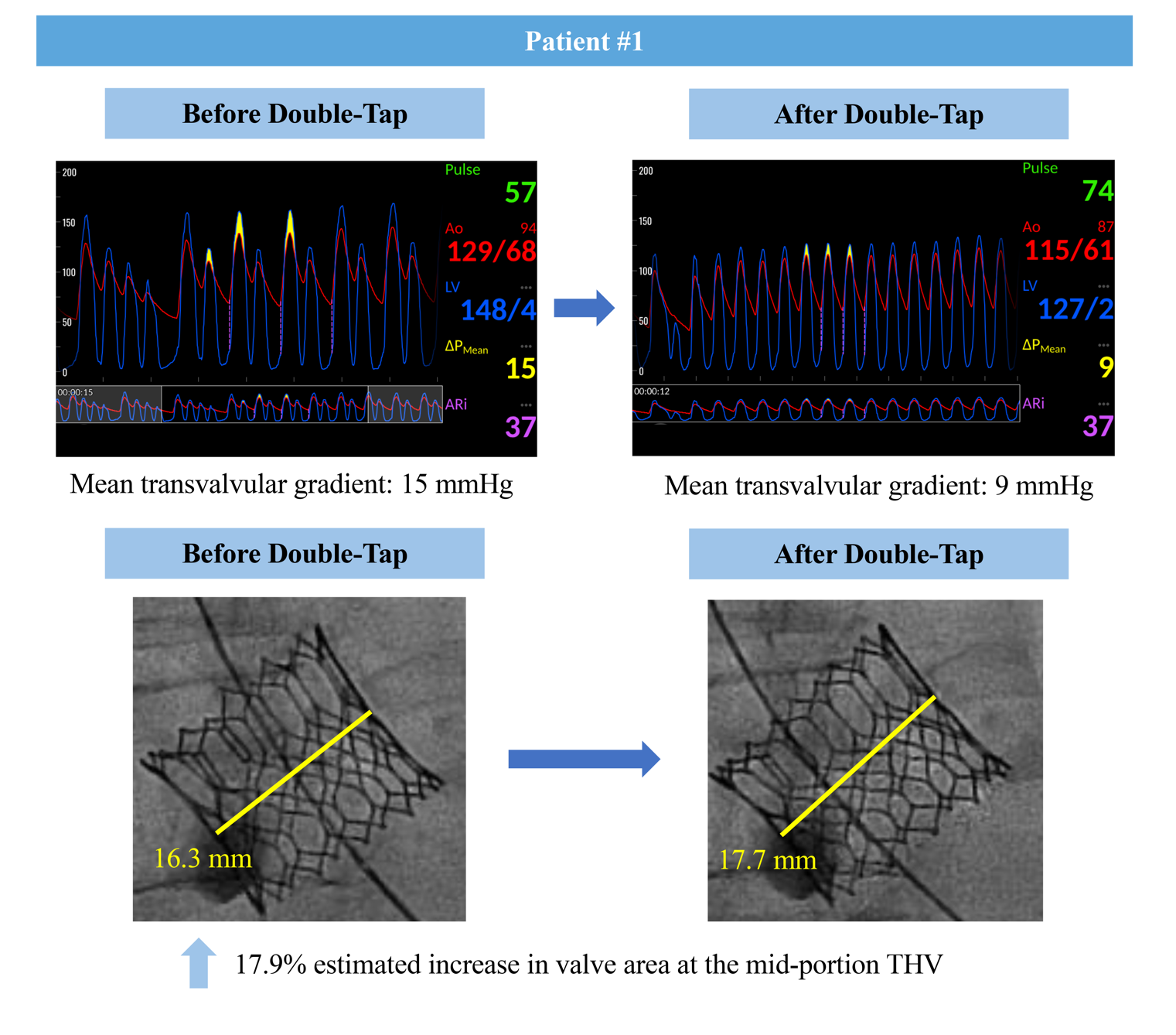


Supplementary Figure 2


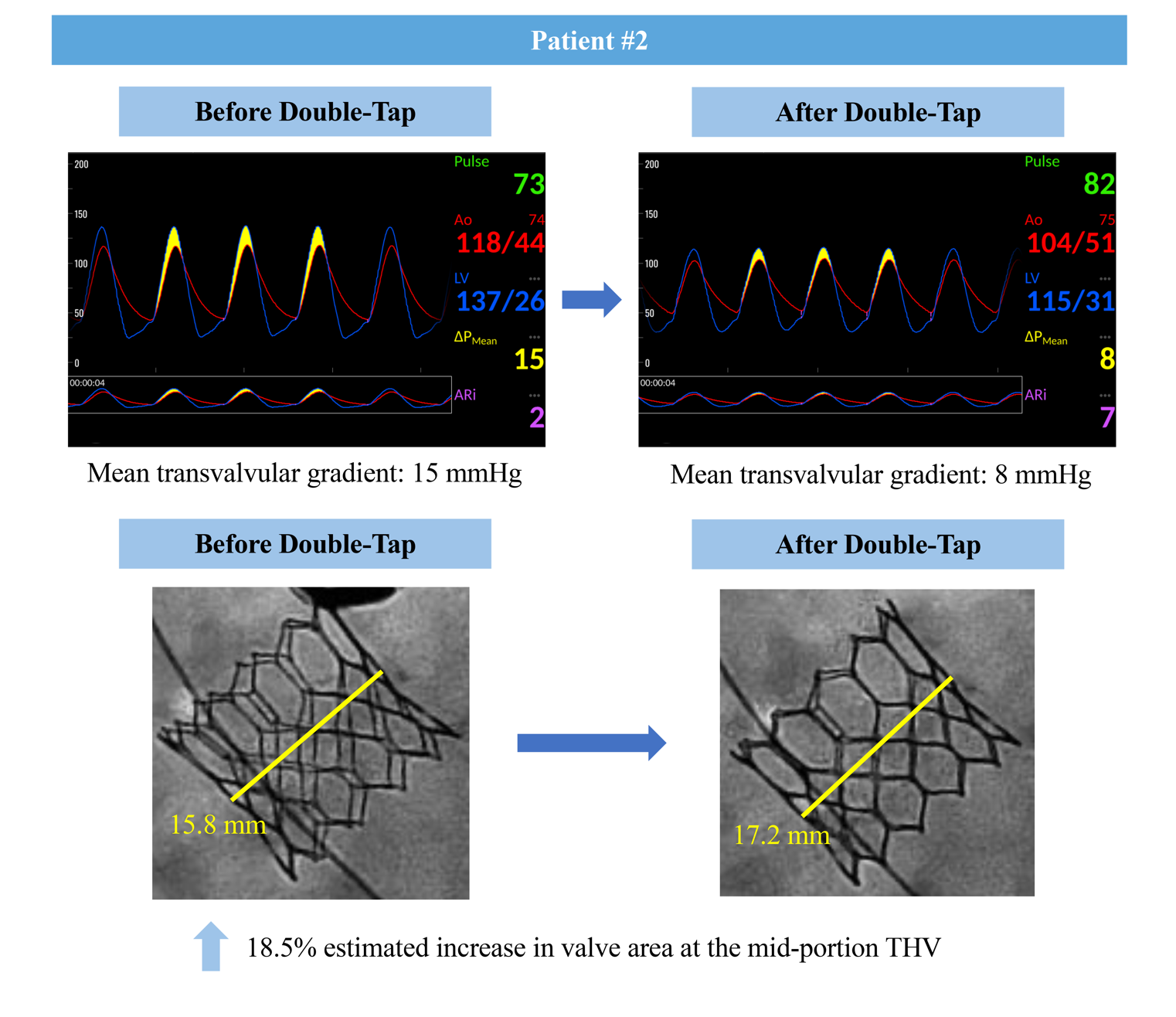


Supplementary Figure 3


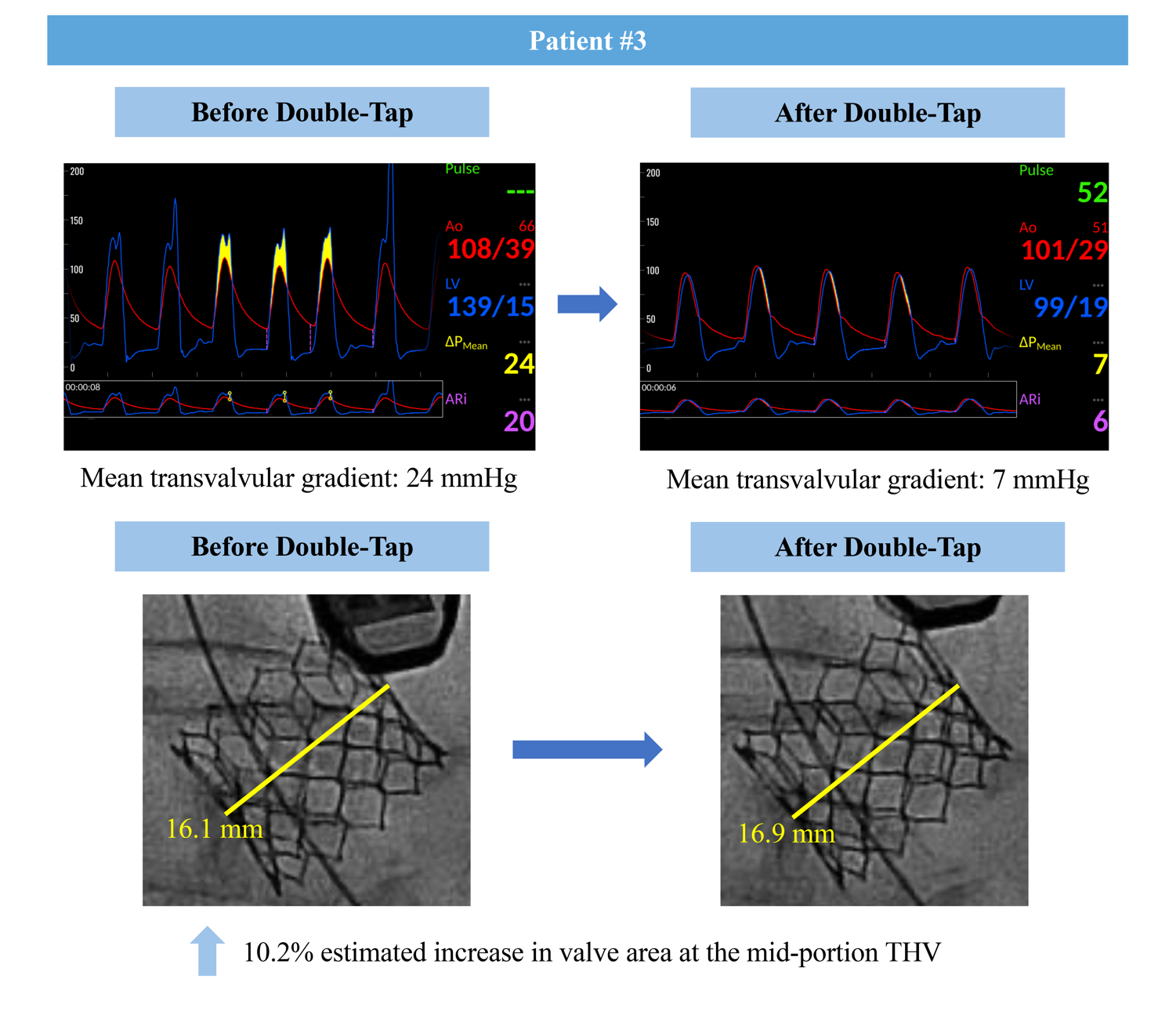


Supplementary Figure 4


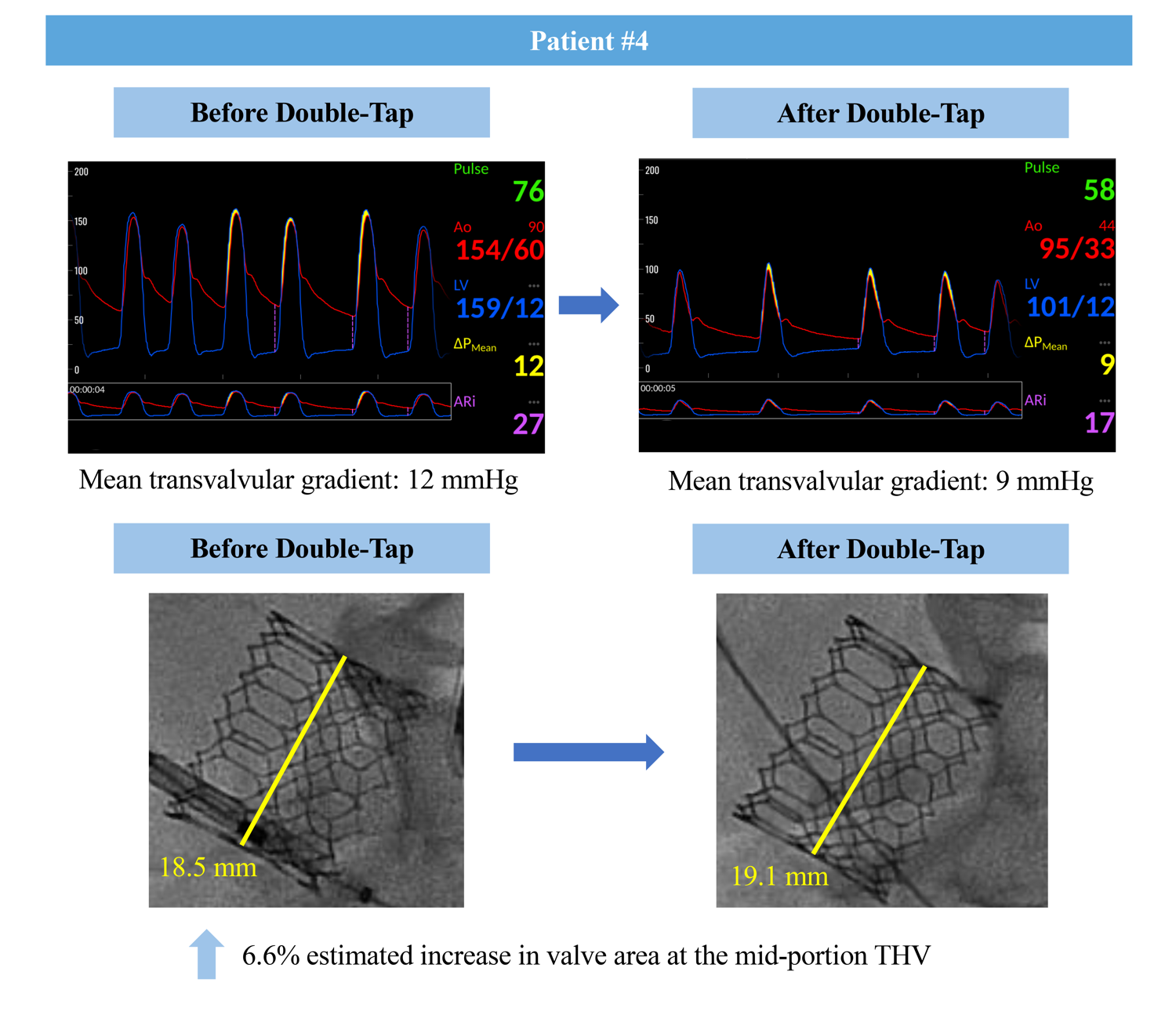


Supplementary Figure 5


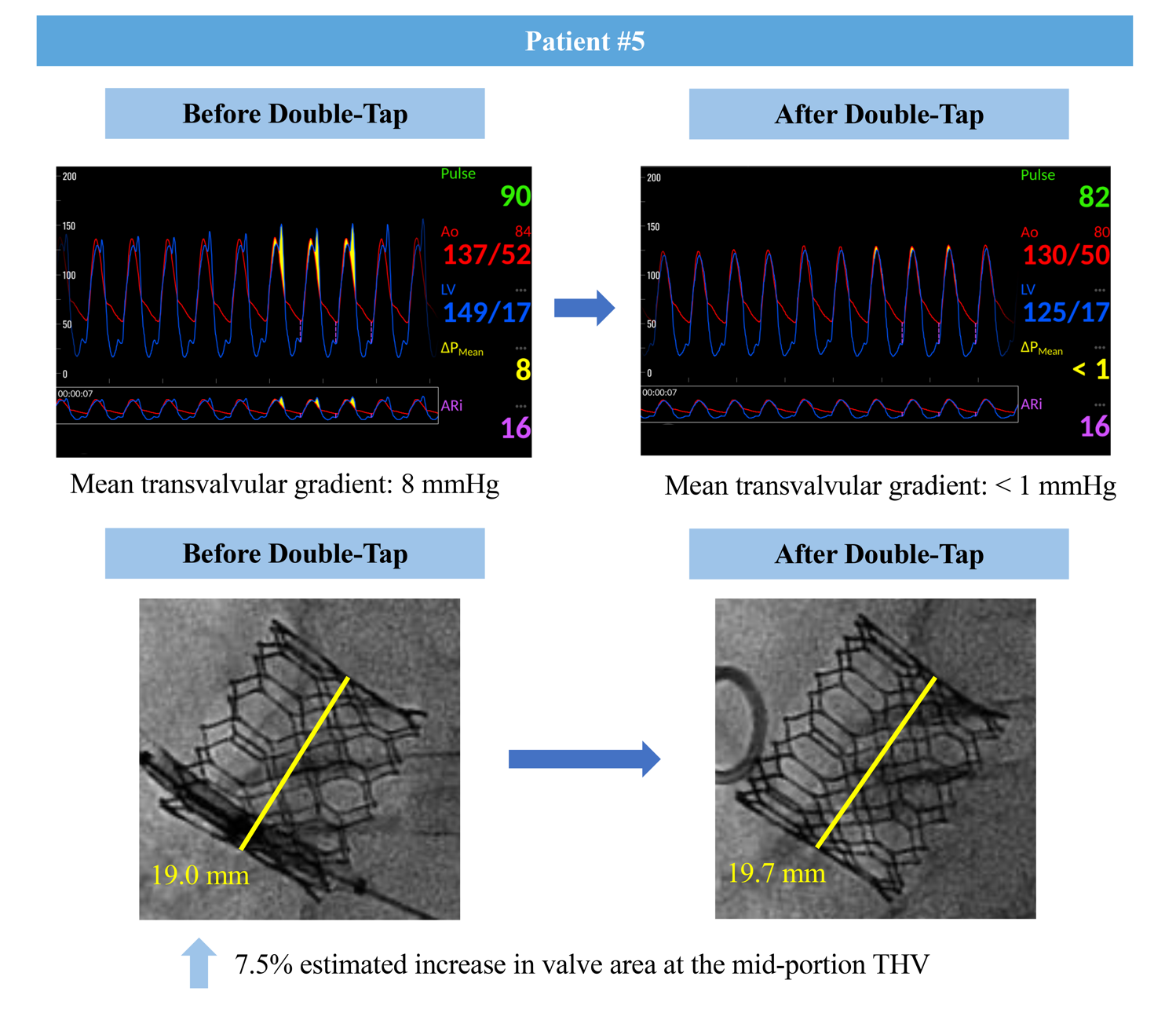


Supplementary Figure 6


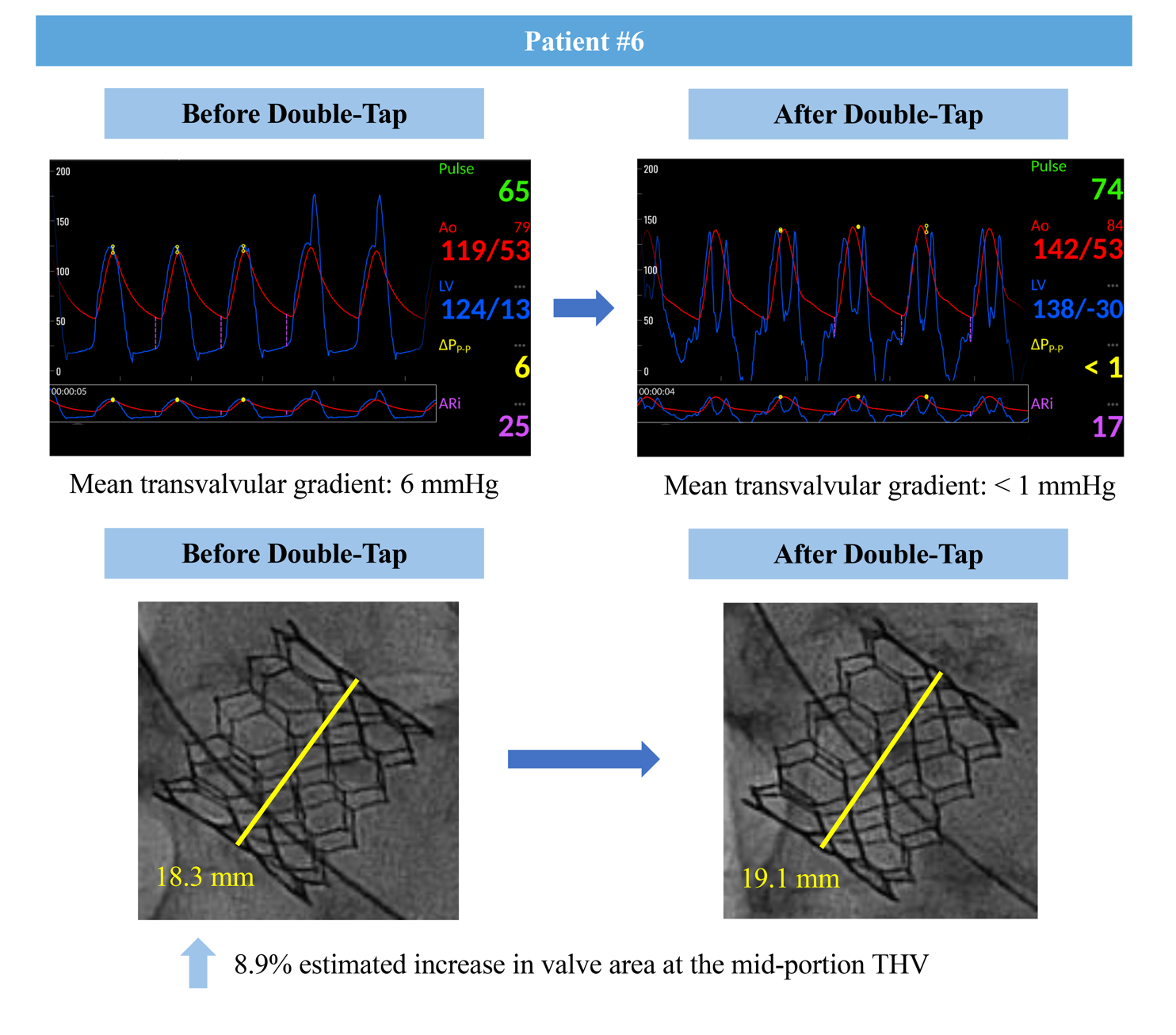

Supplement: Supplemental Figures [file mmc1.docx]
